# Risk factors for labour induction and augmentation: a multicentre prospective cohort study in India

**DOI:** 10.1016/j.lansea.2024.100417

**Published:** 2024-05-07

**Authors:** Tuck Seng Cheng, Farzana Zahir, Solomi V. Carolin, Ashok Verma, Sereesha Rao, Saswati Sanyal Choudhury, Gitanjali Deka, Pranabika Mahanta, Swapna Kakoty, Robin Medhi, Shakuntala Chhabra, Anjali Rani, Amrit Bora, Indrani Roy, Bina Minz, Omesh Kumar Bharti, Rupanjali Deka, Charles Opondo, David Churchill, Marian Knight, Jennifer J. Kurinczuk, Manisha Nair

**Affiliations:** a National Perinatal Epidemiology Unit, Nuffield Department of Population Health, Oxford University, Oxford, UK; b Department of Obstetrics and Gynaecology, Assam Medical College, Dibrugarh, Assam, India; c Department of Obstetrics and Gynaecology, Makunda Christian Leprosy and General Hospital, Karimganj, Assam, India; d Department of Obstetrics and Gynaecology, Dr Rajendra Prasad, Government Medical College, Kangra, Tanda, Himachal Pradesh, India; e Department of Obstetrics and Gynaecology, Silchar Medical College and Hospital, Silchar, Assam, India; f Department of Obstetrics and Gynaecology, Gauhati Medical College and Hospital, Guwahati, Assam, India; g Department of Obstetrics and Gynaecology, Tezpur Medical College, Tezpur, India; h Department of Obstetrics and Gynaecology, Jorhat Medical College and Hospital, Jorhat, Assam, India; i Department of Obstetrics and Gynaecology, Fakhruddin Ali Ahmed Medical College and Hospital, Barpeta, Assam, India; j Department of Obstetrics and Gynaecology, Mahatma Gandhi Institute of Medical Sciences, Sevagram, Maharashtra, India; k Department of Obstetrics and Gynaecology, Banaras Hindu University Institute of Medical Sciences, Varanasi, Uttar Pradesh, India; l Department of Obstetrics and Gynaecology, Sonapur District Hospital, Assam, India; m Department of Obstetrics and Gynaecology, Nazareth Hospital, Shillong, Meghalaya, India; n Department of Obstetrics and Gynaecology, Sewa Bhawan Hospital Society, Chattisgarh, India; o State Institute of Health and Family Welfare, Department of Health & Family Welfare, Government of Himachal Pradesh, India; p MaatHRI Project, Srimanta Sankaradeva University of Health Sciences, Guwahati, Assam, India; q Department of Medical Statistics, London School of Hygiene & Tropical Medicine, London, UK; r Department of Obstetrics and Gynaecology, The Royal Wolverhampton NHS Trust, UK; s Research Institute for Healthcare Science, University of Wolverhampton, UK

**Keywords:** Labour induction, Labour augmentation, Clinical conditions, Non-clinical factors

## Abstract

**Background:**

Guidelines for labour induction/augmentation involve evaluating maternal and fetal complications, and allowing informed decisions from pregnant women. This study aimed to comprehensively explore clinical and non-clinical factors influencing labour induction and augmentation in an Indian population.

**Methods:**

A prospective cohort study included 9305 pregnant women from 13 hospitals across India. Self-reported maternal socio-demographic and lifestyle factors, and maternal medical and obstetric histories from medical records were obtained at recruitment (≥28 weeks of gestation), and women were followed up within 48 h after childbirth. Maternal and fetal clinical information were classified based on guidelines into four groups of clinical factors: (i) ≥2 indications, (ii) one indication, (iii) no indication and (iv) contraindication. Associations of clinical and non-clinical factors (socio-demographic, healthcare utilisation and lifestyle related) with labour induction and augmentation were investigated using multivariable logistic regression analyses.

**Findings:**

Over two-fifths (n = 3936, 42.3%, 95% confidence interval [CI] 41.3–43.3%) of the study population experienced labour induction and more than a quarter (n = 2537, 27.3%, 95% CI 26.4–28.2%) experienced augmentation. Compared with women with ≥2 indications, those with one (adjusted odds ratio [aOR] 0.50, 95% CI 0.42–0.58) or no indication (aOR 0.24, 95% CI 0.20–0.28) or with contraindications (aOR 0.12, 95% CI 0.07–0.20) were less likely to be induced, adjusting for non-clinical characteristics. These associations were similar for labour augmentation. Notably, 34% of women who were induced or augmented did not have any clinical indication. Several maternal demographic (age at labour, parity and body mass index in early pregnancy), healthcare utilization (number of antenatal check-ups, duration of iron-folic acid supplementation and individuals managing childbirth) and socio-economic factors (religion, living below poverty line, maternal education and partner’s occupation) were independently associated with labour induction and augmentation.

**Interpretation:**

Although decisions about induction and augmentation of labour in our study population in India were largely guided by clinical recommendations, we cannot ignore that more than a third of the women did not have an indication. Decisions could also be influenced by non-clinical factors which need further research.

**Funding:**

The MaatHRI platform is funded by a Medical Research Council Career Development Award (Grant Ref: MR/P022030/1) and a Transition Support Award (Grant Ref: MR/W029294/1).

**Research in context**

**Evidence before this study**

A search of published literature until December 01, 2023 showed that while studies have explored the influence of some maternal and fetal risk factors on the decision to induce or augment labour, there is a lack of comprehensive understanding about the role of clinical indications and/or contraindications and non-clinical factors in influencing the decisions, especially in settings with widespread utilization of the labour induction/augmentation.

**Added value of this study**

Our study identified substantial rates of labour induction and/or augmentation in a large-scale multicentre prospective cohort study in India.

In contrast to a study from the UK, we found that women with one or no clinical indication or with contraindications were less likely to be induced or augmented, compared with those with at least two indications.

However, a third of the women who were induced and/or augmented had no clinical indication based on guidelines.

In addition, several non-clinical factors including maternal demographic, healthcare utilisation and socio-economic factors were independently associated with induction or augmentation of labour.

**Implications of all the available evidence**

Our findings highlight the need to understand the complex influence of clinical need and socio-demographic factors on labour induction/augmentation in the context of risk and safety.

Further research is needed to understand the underlying drivers influencing the decision to induce or augment labour, including women’s choice, clinicians’ unconscious bias and awareness of and adherence to guidelines.

## Introduction

Globally, a maternity care crisis has been characterized by the soaring rate of caesarean sections^1^ but now also includes the rapidly growing use of induction and augmentation of labour. Labour induction and augmentation are usually recommended when the continuation of a pregnancy or prolongation of labour is judged to pose more of a clinical risk to maternal and/or fetal health than if the pregnancy were to end. The decision to induce or augment labour has to be balanced with its potential adverse maternal and neonatal outcomes.^2^, ^3^, ^4^, ^5^, ^6^ Several national and international clinical guidelines for labour induction have been established.^7^, ^8^, ^9^, ^10^ While these guidelines identify both maternal and fetal clinical conditions as potential indications for induction/augmentation, there are variations in the specific indications and contraindications within them.^7^^,^^10^ Some additionally suggest offering pregnant women the option to decide on labour induction/augmentation after providing them with relevant information.^7^^,^^10^ This is consistent with the modern practice of shared decision making, enabling maternal autonomy through information provision and choice. Therefore, there may be clinical and non-clinical factors influencing the decisions for labour induction and augmentation.

Several studies have explored the influence of maternal and fetal risk factors on the decision to induce^2^^,^^4^^,^^11^, ^12^, ^13^, ^14^, ^15^, ^16^ or augment labour.^17^^,^^18^ These studies primarily focused on individual maternal indications such as hypertension and diabetes in pregnancy with little consideration given to wider maternal and fetal factors for a more comprehensive understanding of the decision-making process. They also failed to account for the range of clinical opinion as to what are absolute and what are relative contraindications. Grade 3 and 4 placenta praevia are absolute contraindications to induction, whereas breech presentation and two or more previous caesarean sections would trigger a range of responses from clinicians. Moreover, previous studies analysed only a limited number of socio-demographic characteristics such as maternal age and education in influencing decision-making for labour induction/augmentation. The majority of studies are also limited to Western countries (i.e. Belgium, Canada, United Kingdom, United States of America and Poland),^11^, ^12^, ^13^, ^14^^,^^16^^,^^18^ while other studies were performed by continents (i.e. Latin America, Africa and Asia)^2^^,^^4^^,^^15^ rather than in individual countries except for one in Nepal.^17^

In India, maternal morbidity and mortality represents a major public health burden. Every year, nearly 30 million women become pregnant with 5 million facing life-threatening complications.^19^ More worryingly, India records over 45,000 maternal deaths annually, ranking it as the country with the second highest number of maternal deaths worldwide.^20^ The World Health Organization (WHO) Global Survey of 20 Indian facilities in 2007–2008 estimated that 12.8% of pregnant women had an induction of labour,^4^ but there is a lack of more contemporary data. Our study uses data from a multicentre large-scale prospective cohort study^19^ to provide a comprehensive insight into induction and augmentation of labour in pregnant women in India.^19^ The specific objectives of this secondary analysis were to estimate the rates of labour induction and augmentation, and examine the influence of maternal and fetal clinical conditions, socio-demographic, lifestyle and healthcare utilization factors on the decision to induce and/or augment labour.

## Methods

### Data source and study population

We used data from a prospective cohort study conducted through the Maternal and perinatal Health Research collaboration, India (MaatHRI).^19^ MaatHRI is a hospital-based collaborative research platform established in September 2018 to undertake large-scale epidemiological research to improve maternal and perinatal health in a setting with a high burden of maternal mortality and morbidity. To date, this platform includes a network of 16 tertiary and community hospitals across six states in India, namely Assam, Meghalaya, Chhattisgarh, Uttar Pradesh, Himachal Pradesh and Maharashtra.

In this cohort study, pregnant women were enrolled during their third trimester of pregnancy (≥28 weeks of gestation) between January 2018 and August 2023 from 13 of these hospitals (10 Government and 3 private), which participate in epidemiological studies.^21^ The remaining three hospitals have limited staff and resources and are only able to participate in the repeated monthly surveys of severe maternal complication^22^ and not in any other studies. Women were eligible for recruitment in the primary study if they were at least 18 years of age and in the third trimester of pregnancy, planned a vaginal birth in the participating hospital and provided written informed consent. Those who planned an elective caesarean section were not eligible. All recruited pregnant women were followed-up during labour and childbirth and up to 48 h postpartum.

The study was performed in accordance with the principles of the Declaration of Helsinki. Ethics approvals were obtained from the institutional review boards of each coordinating Indian institution, the Government of India’s Health Ministry’s Screening Committee, the Indian Council of Medical Research, New Delhi and the Oxford Tropical Research Ethics Committee (OxTREC), University of Oxford, UK.

### Baseline and follow-up assessments

At the recruitment visit, written informed consent was obtained from all participants including consent for follow-up during and after childbirth. Questionnaires were used by research nurses to collect information face-to-face from pregnant women about their own and their partner’s socio-demographic status and lifestyle factors. Information about the current pregnancy, obstetric and pre-existing medical histories were obtained from the medical records of participants. At the follow-up visit, additional consent was not obtained and further questionnaires were not administered. Only details about labour and childbirth, and pregnancy and infant outcomes were abstracted from medical records. The protocol for data collection from participants and from medical records was standardised across all participating hospitals. All sites use standard uniform definitions and forms, and all research nurses are trained to extract reliable data from medical records.

### Maternal and household characteristics

Information about both clinical and non-clinical factors^23^ were analysed in the study. These included maternal and fetal clinical conditions in current pregnancy, maternal demographic and health characteristics (i.e. age at labour, parity, body mass index (BMI) in early pregnancy, gestational weight gain, previous pregnancy problems, pre-existing medical problems), healthcare utilization indicators (i.e. number of antenatal check-ups, duration of iron-folic acid (IFA) supplementation, individuals managing childbirth), lifestyle factors (i.e. adverse lifestyles such as smoking, alcohol consumption, chewing betel nut or tobacco) and household socio-economic characteristics (i.e. religion, residence, living below poverty line (BPL), maternal education, partner’s occupation).

The maternal and fetal clinical conditions were classified into four groups based on guidelines for labour induction from the WHO,^24^ the National Institute for Health and Care Excellence (NICE),^25^ the American College of Obstetricians and Gynecologists (ACOG),^26^ and the Federation of Obstetric and Gynecological Societies of India (FOGSI)^27^ and the National Health Mission (NHM) in India.^8^^,^^9^^,^^28^ These sources were selected because there are no standard guidelines used across the study hospitals and clinicians reported using one or more of these guidelines or conducting induction and/or augmentation of labour based on clinical judgement of the condition of the pregnant woman and the fetus. The classification was used to generate a categorical variable ‘induction-related clinical indications’, encompassing (i) two or more clinical indications, (ii) one clinical indication, (iii) no clinical indications and (iv) contraindication/s for induction. The clinical indications are detailed in Supplementary Table S1. Contraindications for induction, accepting that there is some variation of opinion, were those with specific placental problems such as placenta praevia, ≥2 previous caesarean sections and fetal malpresentation including breech presentation (Supplementary Table S1), regardless of any aforementioned indication. Women with pre-existing or current pregnancy problems that were neither indications nor a contraindication, and those without any health issues were combined as “No indication” for purposes of the analysis.

Several factors recorded as discrete variables were categorised based on clinical relevance to facilitate interpretations and evaluation of non-linear associations with outcomes. These variables were parity defined as number of completed pregnancies at ≥28 weeks (0, 1, 2–4, ≥5; respectively representing nulliparity, primiparity, multiparity and grand multiparity^29^), number of antenatal check-ups (0–2, 3, 4, ≥5 visits; where the cohort population median is three visits and the WHO recommends at least four visits^30^) and duration of IFA supplementation (none, <100, 100–179, ≥180 days; where national initiative previously recommended supplementation for at least 100 days^31^ and recently recommends at least 180 days^32^).

Adverse lifestyle factors were determined based on responses to questions on smoking status, tobacco consumption, chewing betel nut and alcohol consumption and classified into three categories: i) ‘never’ if all responses were ‘never’, ii) ‘past’ if any response was either ‘gave up prior to pregnancy’ or ‘gave up during pregnancy’ and iii) ‘current’ if any response was ‘current’. The responses were collapsed into one group because almost all reported ‘never’ to smoking (99.1%), and consumptions of tobacco (93.6%) and alcohol (98.4%), and 66.4% reported ‘never’ to chewing betel nut.

Maternal weight in early pregnancy (centred at 10 weeks of gestation) and gestational weight gain per week were estimated from weight measures at the first antenatal visit and during the recruitment visit from medical records using mixed-effects linear regression models with random effects at hospital level^33^ and linear regressions at individual level, respectively. We classified the calculated maternal BMI^34^ in early pregnancy into four categories based on Asian cut-offs^35^: i) underweight (<18.5 kg/m^2^), ii) normal weight (18.5–22.9 kg/m^2^), iii) overweight (23–24.9 kg/m^2^) and iv) obese (≥25 kg/m^2^). For each BMI category in early pregnancy, the gestational weight gain per week was categorized as i) within, ii) below or iii) above the Institute of Medicine guidelines (underweight: 0.44–0.58 kg; normal weight: 0.35–0.50 kg; overweight: 0.23–0.33 kg; obese: 0.17–0.27 kg).^36^

The partner’s (99% married; 1% single) occupation was classified into four levels based on the National Classification of Occupations 2015 in India^37^: i) unemployed, ii) partly skilled and unskilled, iii) skilled manual and non-manual and iv) professional, managerial and technical.

Since this cohort study spanned both pre-pandemic and the entire period of Coronavirus pandemic (COVID-19), the year of childbirth was used to categorise time-period of labour into: i) pre-COVID-19 pandemic (2018–2019), ii) during COVID-19 pandemic (2020–2022) and iii) post-COVID-19 pandemic (2023–2024).

### Induction and augmentation of labour

Information on labour induction and augmentation were collected from medical records which to our knowledge have reasonably accurate information. Furthermore, the collaborating site obstetricians, who are co-authors of the paper, confirmed the data if any discrepancies were found. These data fields were mandatory and so there was no missing information. We were unable to collect information from women who were lost to follow-up. Status concerning labour induction or augmentation were separately recorded in two binary variables (Yes or No). Women who received both interventions are not mutually exclusive and were included in both variables. The methods of labour induction were categorised into three groups: i) mechanical only—if amniotomy, balloon catheter and/or membrane sweeping were conducted without any pharmacological agent, ii) pharmacological only—if misoprostol, oxytocin and/or other prostaglandins were used without any mechanical method, and iii) combination—if both mechanical and pharmacological methods were used. Similarly, the methods of labour augmentation were categorised into: i) mechanical only—if only amniotomy was conducted, ii) pharmacological only—if only oxytocin and/or other prostaglandins were used, and iii) combination—if both mechanical and pharmacological methods were used.

### Statistical analyses

Descriptive analyses were performed to summarise frequencies and proportions of labour induction and augmentation, their methods and clinician-reported indications. Maternal and household characteristics were compared between binary status of labour induction and augmentation, using chi-squared tests for categorical variables and t-tests for continuous variables.

To investigate the associations between clinical and non-clinical factors and labour induction or augmentation, three logistic regression models were computed for each outcome, with sequential addition of variables based on a theoretical framework of the decision-making process for induction and augmentation (Supplementary Fig. S1). Model 1 included only the ‘induction-related clinical indications’ variable. Model 2 additionally included maternal demographic, medical and obstetric characteristics. Model 3 expanded Model 2 by further adding healthcare utilization and lifestyle factors, and household socio-economic characteristics. A sensitivity analysis for labour augmentation was conducted by including labour induction status in Model 3. Another sensitivity analyses for labour induction and augmentation were performed by adding COVID-periods in Model 3 to adjust for any potential effect of the COVID-19 pandemic on management of labour. All models were then compared by assessing the ‘proportions of variance explained’ (R^2^) to estimate variability accounted for by the risk factors and by estimating the area under the receiver operating characteristics curves (AUROC) to assess the risk prediction of each model.

If an association between women’s socio-economic background and labour induction or augmentation was found, we further examined whether specific population groups were different by labour induction/augmentation as per clinical guidelines. To do this, we re-categorised the ‘induction-related clinical indications’ variable into three groups: i) no induction/augmentation ii) induction/augmentation with ≥1 clinical indication and iii) induction/augmentation with no-/contra-indications, and tested their associations with socio-economic factors using multivariable multinomial logistic regressions, adjusting for maternal demographic, medical and obstetric characteristics, healthcare utilization and lifestyle factors.

For all regression analyses, missingness or the unknown category in most variables were treated as missing values given their low proportions (<6%). The unknown category for BPL status was treated as a separate group considering that it was reported by a high proportion (16.4%) of participants.

All statistical analyses were conducted using Stata 17.0 (StataCorp. 2021. Stata Statistical Software: Release 17. College Station, TX: StataCorp LLC) and all associations were considered to be significant at a two-tailed p value of <0.05. Findings from regression analyses were presented as odds ratios (OR) with 95% confidence intervals (CI). We also estimated and reported risk ratios (RR) with 95% CI^38^ for our primary analyses for comparisons.

### Role of the funding source

The funders had no role in the study design, data collection, data analysis and interpretation or writing of the report. The corresponding author had full access to all data in the study and had final responsibility for the decision to submit for publication.

## Results

### Study characteristics

A total of 9420 pregnant women were recruited, of whom 89 (∼1%) were lost to follow-up and one died before childbirth. After further excluding 25 women who later opted for elective caesarean sections, 9305 women were included in the analysis. More than 40% of these women had labour induced (n = 3936, 42.3%, 95% CI: 41.3–43.3%), and in over a quarter of the women labour was augmented (n = 2537, 27.3%, 95% CI: 26.4–28.2%); with nearly one-fifth having both labour induction and augmentation (n = 1789, 19.2%; 95% CI: 18.4–20.0%) (Supplementary Fig. S2). The main clinician-recorded indications (not mutually exclusive) for induction of labour were fetal compromise (n = 727, 18.5%), hypertensive disorders in pregnancy (n = 640, 16.3%) and term pregnancy (n = 498, 12.7%), and for labour augmentation were fetal compromise (n = 788, 31.1%) and labour progression issues (n = 602, 23.7%), see Supplementary Table S2. The most common methods used for labour induction or augmentation were pharmacological only (induction: n = 2316, 58.8%; augmentation: n = 1745, 68.8%), followed by mechanical only (induction: n = 934, 23.7%; augmentation: n = 555, 21.9%) and finally a combination (induction: n = 686, 17.4%; augmentation: n = 237, 9.3%).

### Factors associated with labour induction and augmentation

Table 1 shows the comparisons of maternal and household characteristics among included women with and without labour induction or augmentation. Among women with labour induction and/or augmentation (n = 4684), about 33% (n = 1560) had no clinical indication and 0.7% (n = 35) had a contraindication. Within this ‘contraindication’ sub-group, the majority had malpresentation (n = 20), followed by placental problems (n = 13) and 2–3 previous caesarean sections (n = 2). A majority of them eventually had an emergency caesarean section (n = 26, 74.3%). In general, most maternal and household characteristics were weakly correlated between each other (Spearman correlation, ρ < 0.3), except for age and parity where the correlation was moderate (ρ = 0.443). Partner’s education level was moderately correlated with their own occupation (ρ = 0.390) and also highly correlated with women’s education level (ρ = 0.740), therefore the variable was excluded from the analysis. Also, both women’s marital status and occupation were not included in the analysis due to the low proportions of being single (0.1%) and employed (1.6%).

**Table 1:** Characteristics of included participants with and without labour induction or augmentation in a prospective study in India.

Maternal and household characteristics	No induction	Induced labour	p value	No augmentation	Augmented labour	p value
	N = 5369	N = 3936		N = 6768	N = 2537	
	mean (SD)	mean (SD)		mean (SD)	mean (SD)	
Age at labour (years)	24.85 (4.40)	24.65 (4.50)	0.033	24.73 (4.34)	24.85 (4.72)	0.26
	n (%)	n (%)		n (%)	n (%)	

Induction-related clinical indications			<0.001			<0.001
≥2 indications	466 (8.7)	914 (23.2)		810 (12.0)	570 (22.5)	
1 indication	1911 (35.6)	1824 (46.3)		2563 (37.9)	1172 (46.2)	
No indication	2886 (53.8)	1171 (29.8)		3277 (48.4)	780 (30.7)	
Contraindications	106 (2.0)	27 (0.7)		118 (1.7)	15 (0.6)	
Parity			<0.001			<0.001
0	3278 (61.1)	2963 (75.3)		4435 (65.5)	1806 (71.2)	
1	1391 (25.9)	572 (14.5)		1565 (23.1)	398 (15.7)	
2–4	656 (12.2)	373 (9.5)		729 (10.8)	300 (11.8)	
≥5	44 (0.8)	28 (0.7)		39 (0.6)	33 (1.3)	
BMI categories in early pregnancy			<0.001			<0.001
Underweight (<18.5 kg/m^2^)	1564 (29.1)	878 (22.3)		1698 (25.1)	744 (29.3)	
Normal weight (18.5–22.9 kg/m^2^)	2752 (51.3)	2349 (59.7)		3720 (55.0)	1381 (54.4)	
Overweight (23–24.9 kg/m^2^)	492 (9.2)	364 (9.2)		649 (9.6)	207 (8.2)	
Obesity (≥25 kg/m^2^)	309 (5.8)	221 (5.6)		433 (6.4)	97 (3.8)	
Missing	252 (4.7)	124 (3.2)		268 (4.0)	108 (4.3)	
Gestational weight gain classifications			<0.001			<0.001
Within guidelines	754 (14.0)	588 (14.9)		988 (14.6)	354 (14.0)	
Below guidelines	3468 (64.6)	2752 (69.9)		4435 (65.5)	1785 (70.4)	
Above guidelines	749 (14.0)	432 (11.0)		910 (13.4)	271 (10.7)	
Missing	398 (7.4)	164 (4.2)		435 (6.4)	127 (5.0)	
Previous pregnancy problems			0.027			0.32
No	5097 (94.9)	3717 (94.4)		6408 (94.7)	2406 (94.8)	
Yes	184 (3.4)	170 (4.3)		253 (3.7)	101 (4.0)	
Unknown	88 (1.6)	49 (1.2)		107 (1.6)	30 (1.2)	
Pre-existing medical problems			0.21			<0.001
No	5254 (97.9)	3833 (97.4)		6649 (98.2)	2438 (96.1)	
Yes	114 (2.1)	103 (2.6)		118 (1.7)	99 (3.9)	
Missing	1 (0.0)	0 (0.0)		1 (0.0)	0 (0.0)	
Number of antenatal check-ups			<0.001			<0.001
0–2	1428 (26.6)	592 (15.0)		1580 (23.3)	440 (17.3)	
3	1396 (26.0)	1889 (48.0)		1884 (27.8)	1401 (55.2)	
4	1451 (27.0)	723 (18.4)		1776 (26.2)	398 (15.7)	
≥5	1094 (20.4)	732 (18.6)		1528 (22.6)	298 (11.7)	
Duration of iron-folic acid supplementation (days)			<0.001			<0.001
None	144 (2.7)	228 (5.8)		183 (2.7)	189 (7.4)	
<100	1750 (32.6)	1803 (45.8)		2053 (30.3)	1500 (59.1)	
100–179	2839 (52.9)	1202 (30.5)		3439 (50.8)	602 (23.7)	
≥180	636 (11.8)	703 (17.9)		1093 (16.1)	246 (9.7)	
Individuals managing childbirth			<0.001			<0.001
Auxiliary nurse midwife/nurse	2839 (52.9)	1352 (34.3)		3088 (45.6)	1103 (43.5)	
Resident doctor	2210 (41.2)	2365 (60.1)		3248 (48.0)	1327 (52.3)	
Obstetrician	121 (2.3)	190 (4.8)		216 (3.2)	95 (3.7)	
Others^a^	23 (0.4)	3 (0.1)		15 (0.2)	11 (0.4)	
Missing	176 (3.3)	26 (0.7)		201 (3.0)	1 (0.0)	
Adverse lifestyles (smoking, consumption of tobacco, betel nut or alcohol)			<0.001			<0.001
Never	3617 (67.4)	2416 (61.4)		4543 (67.1)	1490 (58.7)	
Past	226 (4.2)	121 (3.1)		270 (4.0)	77 (3.0)	
Current	1526 (28.4)	1399 (35.5)		1955 (28.9)	970 (38.2)	
Religion			<0.001			<0.001
Hindu	4271 (79.5)	2589 (65.8)		5330 (78.8)	1530 (60.3)	
Muslim	633 (11.8)	1154 (29.3)		994 (14.7)	793 (31.3)	
Christian	225 (4.2)	155 (3.9)		182 (2.7)	198 (7.8)	
Sikh/others	240 (4.5)	38 (1.0)		262 (3.9)	16 (0.6)	
Residence			<0.001			<0.001
Rural	4698 (87.5)	3254 (82.7)		5695 (84.1)	2257 (89.0)	
Suburb/urban	671 (12.5)	682 (17.3)		1073 (15.9)	280 (11.0)	
Living below poverty line (BPL)			<0.001			<0.001
BPL certificate/self-certified	2203 (41.0)	2457 (62.4)		2748 (40.6)	1912 (75.4)	
Not BPL	2074 (38.6)	1047 (26.6)		2720 (40.2)	401 (15.8)	
Unknown	1092 (20.3)	432 (11.0)		1300 (19.2)	224 (8.8)	
Woman’s education			<0.001			<0.001
Illiterate	647 (12.1)	270 (6.9)		739 (10.9)	178 (7.0)	
≤5th class	729 (13.6)	1194 (30.3)		1085 (16.0)	838 (33.0)	
6–12th class	2952 (55.0)	1953 (49.6)		3639 (53.8)	1266 (49.9)	
≥12th class	1017 (18.9)	510 (13.0)		1273 (18.8)	254 (10.0)	
Unknown	24 (0.4)	9 (0.2)		32 (0.5)	1 (0.0)	
Partner’s occupation			<0.001			<0.001
Unemployed	186 (3.5)	402 (10.2)		474 (7.0)	114 (4.5)	
Partly skilled and unskilled	1567 (29.2)	1215 (30.9)		1765 (26.1)	1017 (40.1)	
Skilled manual and non-manual	3334 (62.1)	2138 (54.3)		4216 (62.3)	1256 (49.5)	
Professional, managerial and technical	275 (5.1)	180 (4.6)		306 (4.5)	149 (5.9)	
Non applicable/missing	7 (0.1)	1 (0.0)		7 (0.1)	1 (0.0)	
COVID-19 period			<0.001			<0.001
2018–2019 (pre-COVID-19 pandemic)	1425 (26.5)	754 (19.2)		1686 (24.9)	493 (19.4)	
2020–2022 (during COVID-19 pandemic)	3582 (66.7)	2828 (71.8)		4591 (67.8)	1819 (71.7)	
2023–2024 (post-COVID-19 pandemic)	362 (6.7)	354 (9.0)		491 (7.3)	225 (8.9)	

SD, standard deviation; n, number; %, percentage; COVID; Coronavirus disease 2019.

a Family members, postgraduate trainee and community health workers.

**Fig. 1:**
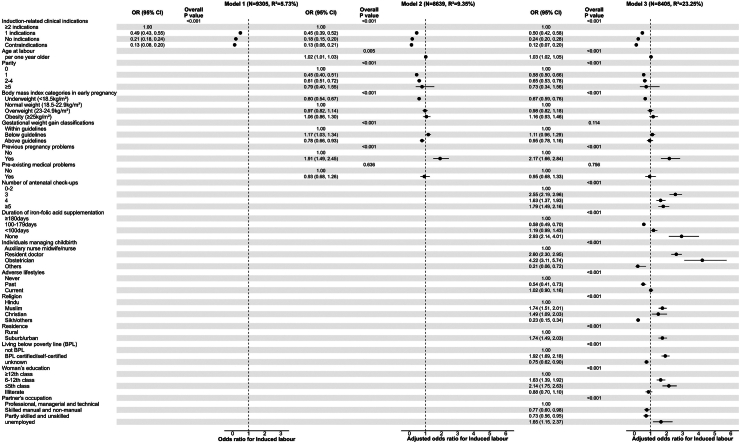
Associations of maternal and household characteristics with labour induction in a prospective study in India.

Fig. 1 shows the associations of maternal and household characteristics with labour induction. Compared with women who had two or more induction-related clinical indications, those with one or no indication or with contraindication were significantly less likely to undergo induction of labour. These findings remained consistent after adjustment for other maternal and household characteristics in all three models (Model 3–one indication: adjusted odds ratio (aOR): 0.50, 95% CI: 0.42, 0.58; no indication: aOR: 0.24, 95% CI: 0.20, 0.28; contraindication: aOR: 0.12, 95% CI: 0.07, 0.20). When simultaneously considering clinical indications, and maternal demographic and health characteristics (Model 2), primiparity and multiparity of 2–4 (vs. nulliparity), underweight (vs. normal weight) in early pregnancy and gestational weight gain above (vs. within) guidelines were less likely to be associated with induced labour. The odds of induction increased by 2% per year increase in age of the pregnant women at labour. The odds of induction was also higher for women with a gestational weight gain below the recommended guidelines and a history of previous pregnancy problems. With the exception of gestational weight gain, these associations remained significant even after adjusting for healthcare utilization, lifestyle, and household socio-economic factors in Model 3. Furthermore, women had a higher odds of induction if they had a higher number of antenatal check-ups (≥3 vs. 0–2), if childbirth was managed by a resident doctor and obstetrician (vs. auxiliary nurse midwife/nurse) and if they did not receive any IFA supplementation (vs. ≥180 days of supplementation) during pregnancy. A lower level of education (≤5th and 6–12th vs. ≥12th class), partner being unemployed (vs. in professional employment), BPL certified/self-certified (vs. not BPL), belonging to a minority religious Muslim or Christian background (vs. Hindu), and living in suburban/urban (vs. rural) areas were associated with increased odds of induction of labour. Similar associations expressed as risk ratios were observed (Supplementary Fig. S3). Overall, the R^2^ for labour induction increased from 5.7% to 23.25% (Fig. 1 and Supplementary Fig. S3), while the AUROCs for predicting labour induction increased from 0.67 to 0.79 (Supplementary Fig. S4), with the addition of non-clinical factors beyond clinical indications for induction.

**Fig. 2:**
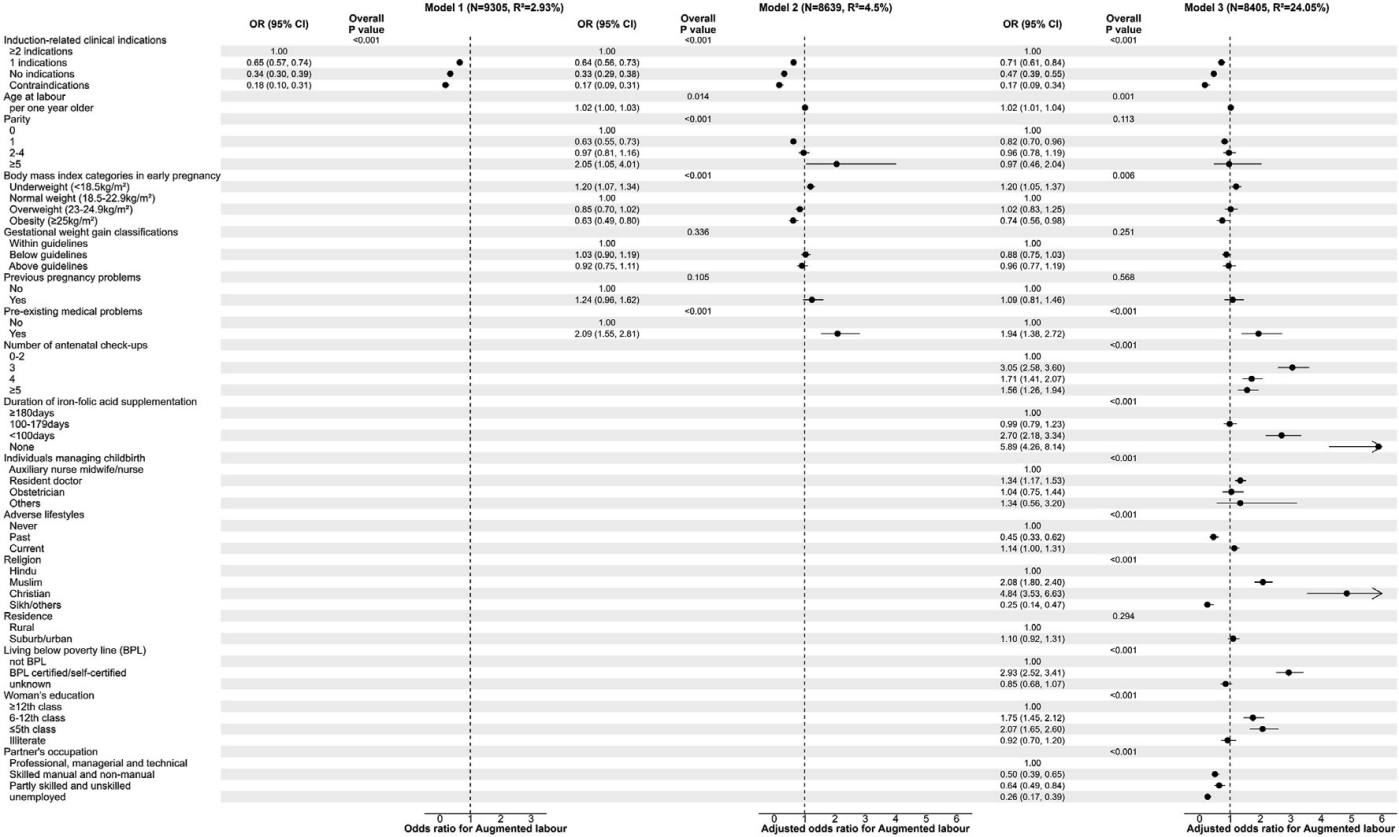
Associations of maternal and household characteristics with labour augmentation in a prospective study in India.

Fig. 2 shows the associations of maternal and household characteristics with labour augmentation. The results were largely similar to those observed for induction of labour. Compared with women who had two or more induction-related clinical indications, those with one indication (aOR: 0.71, 95% CI: 0.61, 0.8 4), no indication (aOR: 0.47, 95% CI: 0.39, 0.55) and contraindications (aOR: 0.17, 95% CI: 0.09, 0.34) were less likely to undergo labour augmentation, after adjustment for other maternal and household characteristics in all models. Also, per year increase in age at labour, being underweight in early pregnancy, having pre-existing medical problems, higher number of antenatal check-ups, lower duration of IFA supplementation, being managed by resident doctor, belonging to a minority religious Muslim or Christian background, BPL certified/self-certified and a having lower level of education were independently associated with higher odds of augmentation (Model 3). These associations were similar when expressed as risk ratios (Supplementary Fig. S5). Upon adding labour induction status into Model 3, the observed associations mostly remained significant and women who had an induction of labour had more than three-fold higher odds of augmentation (Supplementary Fig. S6). Overall, the R^2^ for labour augmentation increased from 2.9% to 24.1% (Fig. 2 and Supplementary Fig. S5), while the AUROCs for predicting labour augmentation increased from 0.62 to 0.83, when maternal and household characteristics were added (Supplementary Fig. S7). The R^2^ increased marginally to 27.4% (Supplementary Fig. S6) but the AUROC remained unchanged (Supplementary Fig. S7) when labour induction was additionally included.

All findings about labour induction and augmentation remained unchanged upon additional adjustment for COVID-periods (Supplementary Fig. S8). Compared with the pre-COVID-19 pandemic, higher odds of induction and augmentation were observed during pandemic study periods. However, the inclusion of the variable COVID-periods into Model 3 had minimal impact on R^2^ values (24.0% for labour induction; 24.1% for labour augmentation). The AUROCs showed little change for induction (0.79, 95% CI: 0.79–0.81) and augmentation (0.81, 95% CI: 0.80–0.82) compared with the main model.

Supplementary Table S3 shows the adjusted associations between socio-economic factors and clinical indications for labour induction and augmentation. Compared with women with no induction or augmentation, women who belonged to minority religious Muslim or Christian background, lived in suburban or urban areas, reported BPL and had a lower level of education were more likely to have labour induction with both ≥1 clinical indications and/or with no-/contra-indication.

## Discussions

In this secondary analysis of a large prospective cohort study of pregnant women from 13 hospitals in India, a substantial rate of labour induction/augmentation was reported. Two out of every five women had labour artificially induced or over a quarter had their labour augmented; with nearly one-fifth having both induction and augmentation. Approximately two-thirds of the methods used for labour induction or augmentation were pharmacological only. Consistent with current guidelines for labour induction,^8^^,^^9^^,^^24^, ^25^, ^26^, ^27^, ^28^ the greater the number of maternal and fetal clinical indications a woman had, the more likely her labour was to be induced. Approximately 1% of women who were induced or augmented had clinical contraindications and approximately 33% did not have any documented indication. The associations with clinical indications were independent of other non-clinical maternal and household characteristics. In addition, maternal demographic (age at labour, parity and BMI in early pregnancy), healthcare utilization (antenatal check-ups, duration of IFA supplementation and individuals managing childbirth) and socio-economic characteristics (religion, BPL status, maternal education and partner’s occupation) were independently associated with labour induction and augmentation. Most of these associations had a magnitude of at least 10% higher or lower than the reference groups and were therefore considered clinically significant. On further analyses, we found that women living in suburban/urban areas and from disadvantaged and minority socio-economic backgrounds were more likely to undergo induction and/or augmentation if they had clinical indications as well as if they had no indications or contraindications.

Compared with the WHO Global Survey of 20 Indian healthcare facilities in 2007–2008,^4^ the present findings suggest a potential threefold increase in the prevalence of labour induction in India (from 12.8% in the WHO survey to 42.3% in our study) over two decades, with induction using pharmacological agents alone being the predominant method (77.4% in the WHO survey vs. 58.8% in our study). Also, the WHO Global Survey^4^ and our study consistently found that fetal-related complications and hypertensive disorders in pregnancy were the most common indications for induction (representing at least 10% of inductions), and identified similar proportions of induction of labour without any clinical indication as per guidelines^8^^,^^9^^,^^24^, ^25^, ^26^, ^27^, ^28^ (32.1% in WHO survey vs. ∼31% in our study). The increased utilization of induction of labour may be partially explained by improved diagnosis or increase in the prevalence of maternal and/or fetal complications, but this also depends on what is reported in the labour management records.

Our study largely expands previous analyses in other populations,^2^^,^^4^^,^^11^, ^12^, ^13^, ^14^, ^15^, ^16^, ^17^, ^18^ encompassing a comprehensive range of both maternal and fetal clinical factors in relation to labour induction and augmentation. Most studies have individually examined health complications in mothers and/or fetus, and reported associations of various clinical conditions, including hypertensive disorders in pregnancy and non-indications^8^^,^^9^ such as anxiety, depression and urinary tract infection, with labour induction^2^^,^^4^^,^^11^^,^^12^ or augmentation.^17^^,^^18^ One of these studies also showed several contraindications (i.e. placental abruption, placenta praevia, cord prolapse and breech or malpresentation) to be less likely to be associated with labour induction.^11^ Our findings highlight generally good clinical practice in the study hospitals, although a third of the women who were induced or augmented had no clinical indication. A small number of women with contraindications were also induced (n = 27) or augmented (n = 15). These contraindications were identified during antenatal visits or recorded as indications for emergency caesarean section. These observations suggest that clinicians may have potentially missed or not fully considered all pertinent information in medical records, or not adequately assessed women’s and their fetus’s health conditions before labour induction/augmentation. It is also possible that the decision to induce and augment was based on clinical judgement of benefit to the mother and/or the fetus irrespective of whether the labour intervention was necessary as per guidelines. In contrast, a study in the United Kingdom that classified pregnant women into four categories, i.e. no pregnancy complications, pregnancy complications not usually associated with labour induction, pregnancy complications associated with labour induction, and others, observed all complications groups to be associated with labour induction.^16^

Through the analyses of a wide range of non-clinical factors, this study highlighted that several maternal demographic, healthcare utilization and socio-economic factors could influence the decision to induce and/or augment labour. The combination of these non-clinical factors yielded a more comprehensive explanation of variations in labour induction and augmentation, and increased the predictions for labour induction and augmentation, as noted by the increased R^2^ and AUROCs. Similar to our findings, previous studies in the United States of America, United Kingdom, Belgium, and Latin American, African and Asian regions have reported that pregnant women who were in the older age groups,^2^^,^^15^ had lower education level,^13^^,^^15^^,^^16^ more antenatal visits/prenatal care^4^^,^^11^^,^^15^ or childbirth attended by physicians,^11^ or lived in urban areas^15^ tended to undergo induction of labour. Associations with labour augmentation were however mostly different in previous studies with higher odds observed for pregnant women who were younger or nulliparous, or had a higher education level in Nepal^17^ or had a higher BMI in Poland.^18^

Our findings indicate a complex involvement of clinical practices and maternal characteristics, beyond pathologies alone, in influencing the decisions for inducing and augmenting labour. It is possible that clinicians lower their threshold for labour induction/augmentation based on other risk factors due to various reasons, irrespective of clinical indications. Older pregnant women or women with previous pregnancy problems or pre-existing medical problems, from lower socio-economic status or from religious minority backgrounds might be thought to be at a higher risk of adverse maternal and fetal outcomes^39^ and hence more likely to be offered labour induction and/or augmentation. Indeed on further analyses, women from disadvantaged or minority backgrounds were found to undergo induction/augmentation if they had one or more clinical indications, but also if they had no indication or a contraindication. A tendency to intervene despite no clinical indications may arise from pressure by public or institutional expectations, or fear of potential abuse or litigation.^39^ Alternatively, clinicians may be guided by the changes in societal norms with a greater emphasis on shared decision making and a woman’s right to choose, which unintentionally allows certain groups of women leaning towards labour induction/augmentation. Women who came from lower socio-economic status or lived in suburb or urban areas, or belonged to religious minorities may tend to view induction/augmentation as a better option (such as for pain relief) due to poor access to information from unreliable sources such as social media, or based on cultural or religious beliefs.^39^ Again, it is possible that women who had more antenatal visits possibly had higher expectations for labour induction/augmentation.

This is the first large-scale prospective cohort study in India that comprehensively examined the clinical and non-clinical factors influencing decisions for labour induction/augmentation. However, the study was conducted in 13 hospitals in India, which limits the generalizability of our findings to all healthcare facilities in India and other settings. Our findings are conditioned on guidelines^8^^,^^9^^,^^24^, ^25^, ^26^, ^27^, ^28^ from multiple sources to encompass a broader range of potential clinical indications and contraindications for labour induction to minimize misclassification of the sub-categories. Systematic bias may exist if clinicians tended to selectively report relevant clinical indications for women undergoing induced or augmented labour, but we carefully minimized this bias by taking into account all maternal and fetal clinical conditions, which were prospectively ascertained at recruitment and after childbirth from medical records, instead of relying only on clinician-reported indications. The small number of women who had a placental problem but were still induced or augmented might have been misclassified as contraindicated due to insufficient information. These could have been women with minor grade placenta previa but as information on grade of severity was not available, women with any reported placental problems were classified under the contraindication group. Furthermore, contraindications for induction are controversial and mainly provided by the FOGSI but not others.^8^^,^^9^^,^^24^, ^25^, ^26^, ^27^, ^28^ Moreoever, socio-economic characteristics and lifestyles may be subject to reporting bias for various reasons such as social desirability, although these are likely to be nondifferential misclassifications which bias associations towards the null. The status of labour induction and augmentation may have been cross-misclassified as certain methods used to induce or augment labour overlap such as amniotomy^16^ and oxytocin.^40^ However, we have minimized the potential biases by ensuring no duplication in the reported methods of labour induction and augmentation for each woman. Although our analyses included a wide range of maternal healthcare utilization, lifestyle and socio-demographic characteristics, our observed associations may be affected by other unmeasured risk factors such as age at first pregnancy and distance to the health facility. We did not include labour duration in the augmentation analysis models to avoid overadjustment as labour duration could be an outcome of the augmentation process itself. We also cannot rule out the possibility of chance findings as we tested the associations of induction and augmentation with a wide range of non-clinical factors. Although there was a 9.7% reduction in sample size from Model 1 to Model 3 due to complete case analyses, the estimates of associations between models did not change materially. Finally, the increases in R^2^ and AUROCs across the three regressions models may be partly explained by the addition of more variables in the models.

In conclusion, our analyses of pregnant women across multiple hospitals in India identified high proportions of women undergoing labour induction and/or augmentation, predominantly using pharmacological agents. We found that pregnant women with fewer indications and those with contraindications, as per guidelines for labour induction, were less likely to be induced or augmented, suggesting good clinical practice in general. However, we cannot ignore that some women (a third) with no apparent indication or with contraindications were induced and/or augmented. Our findings importantly highlight that non-clinical factors such as maternal healthcare utilization and socio-demographic characteristics, beyond the commonly considered maternal and fetal complications, could potentially influence the decision-making process for labour interventions in India. Future in-depth research is needed to understand the complex influence of clinical need and socio-demographic factors on labour induction/augmentation in the context of risk and safety to enable women to be properly informed about the consequences of labour induction and augmentation. Replicating similar studies in other settings is crucial to determine the extent of non-clinical influences on labour induction and augmentation. This in turn can contribute towards improving local clinical guidelines, empowering women with adequate and appropriate information for decision-making, mitigating clinicians’ unconscious bias and enhancing clinicians’ awareness of and adherence to guidelines, amidst the widespread utilization of the invasive labour induction/augmentation.

## Contributors

TSC and MN conceptualised this secondary data analysis. MN designed the study and developed the methodology. TSC conducted the statistical analysis and wrote the first draft of paper. TSC, CO, DC, SSC and MN interpreted the findings. FZ, SVC, AV, SR, SSC, GD, PM, SK, RM, SC, AR, AB, IR, BM and OKB are collaborators and investigators for the study, and contributed equally to developing the study and led the work in their respective institution. RD is MaatHRI project manager and supervised data collection, entry and compilation. MK and JK are advisors and contributed to developing the study. All authors read and approved the final version of the manuscript. The corresponding author attests that all listed authors meet authorship criteria and that no others meeting the criteria have been omitted.

## Data sharing statement

Data are available upon reasonable request by contacting the corresponding author.

## Declaration of interests

The authors declare that they have no known competing financial interests or personal relationships that could have appeared to influence the work reported in this paper.
