# Supplementary material for: Risk factors for labour induction and augmentation: a multicentre prospective cohort study in India
Source: Lancet Reg Health Southeast Asia. 2024 May 7;25:100417. doi: 10.1016/j.lansea.2024.100417 (PMC11097080; doi:10.1016/j.lansea.2024.100417)
Supplement: Supplementary Figs. S1–S8 Tables S1–S3 [file mmc1.docx]

| **Supplementary Table S1: Maternal and fetal indications and contraindications for induction of labour according to guidelines*** | |
| --- | --- |
| **Indications** | **Contraindications** |
| - Pre-existing or current pregnancy health problems - Pre-existing diabetes mellitus or gestational diabetes mellitus - Hypertension disorders in pregnancy including pre-existing hypertension, pregnancy-induced hypertension, preeclampsia and eclampsia - Antepartum haemorrhage - Premature rupture of membrane - Chorioamnionitis - Oligohydramnios - Obstetric cholestasis - Isoimmunisation - Renal disease - Cardiac problem - Current fetal health problems - Structural defects - Intrauterine demise - Small for gestational age (based on birthweight <2500g) - Post-term pregnancy (≥41 weeks of gestation calculated from last menstrual period) - Multiple gestation | - Specific placental problems such as a major placenta praevia - ≥2 previous caesarean sections - Fetal malpresentation including breech presentation |
| *the World Health Organization,^24^ the National Institute for Health and Care Excellence,^25^ the American College of Obstetricians and Gynecologists,^26^ the Federation of Obstetric and Gynecological Societies of India^27^ and the National Health Mission in India^28^ | |

**Supplementary Figure S1: Theoretical framework of the decision-making process for labour induction and augmentation**

**Healthcare utilization and lifestyle factors**

- number of antenatal check-ups
- duration of iron-folic acid supplementation
- healthcare professional managing childbirth
- adverse lifestyles

**Maternal demographic, medical and obstetric characteristics**

- age at labour
- parity
- body mass index in early pregnancy
- gestational weight gain
- previous pregnancy problems
- pre-existing medical problems

**Induction-related clinical indications**

**Labour induction**

**Labour augmentation**

**Household socio-economic characteristics**

- religion
- residence
- living below poverty line
- maternal education
- husband’s occupation

**Supplementary Figure S2: Flow chart for the study inclusion and characteristics**

9420 recruited

9305 included

- Loss to follow-up (n=89)
- Death before childbirth (n=1)
- Opting for elective caesarean sections after recruitment (n=25)

3936 induced labour

2537 augmented labour

1789 induced and augmented labour

| **Supplementary Table S2: Frequencies of clinician-reported indications for labour induction and augmentation in the study hospitals in India** | | | | | |  |
| --- | --- | --- | --- | --- | --- | --- |
| **Reported indications*** | **Labour induction** | |  | **Labour augmentation** | | |
|  | **N** | **%** |  | **N** | **%** | |
| *Maternal factors* |  |  |  |  |  | |
| Pregnancy-induced hypertension/ hypertension | 588 | 14.94 |  | 28 | 1.10 | |
| Term pregnancy | 498 | 12.65 |  | 222 | 8.75 | |
| Post-term pregnancy | 363 | 9.22 |  | 47 | 1.85 | |
| Premature rupture of membrane | 340 | 8.64 |  | 96 | 3.78 | |
| Oligohydramnios | 184 | 4.67 |  | 6 | 0.24 | |
| Rupture of membrane | 134 | 3.40 |  | 48 | 1.89 | |
| Preterm pregnancy | 114 | 2.90 |  | 36 | 1.42 | |
| Early labour | 51 | 1.30 |  | 3 | 0.12 | |
| Labour pain | 48 | 1.22 |  | 26 | 1.02 | |
| Pre-eclampsia | 44 | 1.12 |  | 7 | 0.28 | |
| Anaemia | 42 | 1.07 |  | 12 | 0.47 | |
| Latent labour | 34 | 0.86 |  | 4 | 0.16 | |
| No labour | 26 | 0.66 |  | 10 | 0.39 | |
| Cholestasis | 16 | 0.41 |  | - | - | |
| Gestational diabetes mellitus/ diabetes | 13 | 0.33 |  | 2 | 0.08 | |
| Previous caesarean sections | 13 | 0.33 |  | - | - | |
| Anhydraminious | 12 | 0.30 |  | - | - | |
| Antepartum haemorrhage | 10 | 0.25 |  | 1 | 0.04 | |
| Eclampsia | 8 | 0.20 |  | 4 | 0.16 | |
| Grand multiparity | 8 | 0.20 |  | - | - | |
| Rhesus negative | 7 | 0.18 |  | - | - | |
| Short stature | 6 | 0.15 |  | - | - | |
| Hypothyroidism | 6 | 0.15 |  | 1 | 0.04 | |
| Polyhydraminious | 5 | 0.13 |  | - | - | |
| Fetal demise in previous pregnancy | 5 | 0.13 |  | - | - | |
| Elderly primigravida | 4 | 0.10 |  | - | - | |
| Multiple gestation | 4 | 0.10 |  | 1 | 0.04 | |
| Oedema | 4 | 0.10 |  | - | - | |
| Meconium stained liquor | 4 | 0.10 |  | 14 | 0.55 | |
| Poor obstetric history | 4 | 0.10 |  | - | - | |
| Haemoglobin E disease | 3 | 0.08 |  | 2 | 0.08 | |
| Uneffaced cervix | 3 | 0.08 |  | 5 | 0.20 | |
| Deteriorating maternal condition | 2 | 0.05 |  | - | - | |
| Chorioamnionitis | 2 | 0.05 |  | 1 | 0.04 | |
| Infertility | 2 | 0.05 |  | - | - | |
| Other maternal factors^†^ | 13 | 0.33 |  | - | - | |
|  |  |  |  |  |  | |
| *Fetal factors* |  |  |  |  |  | |
| Fetal compromise | 727 | 18.47 |  | 788 | 31.06 | |
| Decreased fetal movement | 127 | 3.23 |  | 3 | 0.12 | |
| Low birth weight / IUGR | 46 | 1.17 |  | 6 | 0.24 | |
| Fetal demise | 26 | 0.66 |  | 17 | 0.67 | |
| Fetal distress | 24 | 0.61 |  | - | - | |
| Breech presentation | 8 | 0.20 |  | - | - | |
| Non-reassuring fetal heat rate | 6 | 0.15 |  | 3 | 0.12 | |
| Abnormal Doppler | 4 | 0.10 |  | - | - | |
| Mobile fetal head | 4 | 0.10 |  | 1 | 0.04 | |
| Big baby | 2 | 0.05 |  | 1 | 0.04 | |
| Other fetal factors^‡^ | 2 | 0.05 |  | 1 | 0.04 | |
|  |  |  |  |  |  | |
| *Labour-related factors* |  |  |  |  |  | |
| Prolonged labour / no or slow progress in labour^¥^ | 116 | 2.95 |  | 203 | 8.00 | |
| To accelerate labour^¥^ | 17 | 0.43 |  | 223 | 8.79 | |
| Reported induction failure^¥^ | 11 | 0.28 |  | 10 | 0.39 | |
| Poor or inadequate uterine contraction | - | - |  | 495 | 19.51 | |
| *not mutually exclusive (i.e. women could have >1 indication)  ^†^including cardiac problems, renal problems, placenta problems, abortion, history of abortion, ascites, breathing difficulty, difficulty in childbirth in previous pregnancies, abnormal liver function test, obesity, history of preterm childbirth, high risk pregnancy  ^‡^including birth asphyxia, cephalopelvic disproportion  %, percentages calculated by dividing the number of a particular reported indication separately by total induced (n=3936) or augmented (n=2537) labour  ^¥^Note: These indicate that the woman was already in labour, but as these were mentioned as reasons for induction of labour, we have reported the information as such. | | | | | |  |

**Supplementary Figure S3: Associations of maternal and household characteristics with labour induction in a prospective study in India, expressed as risk ratios**


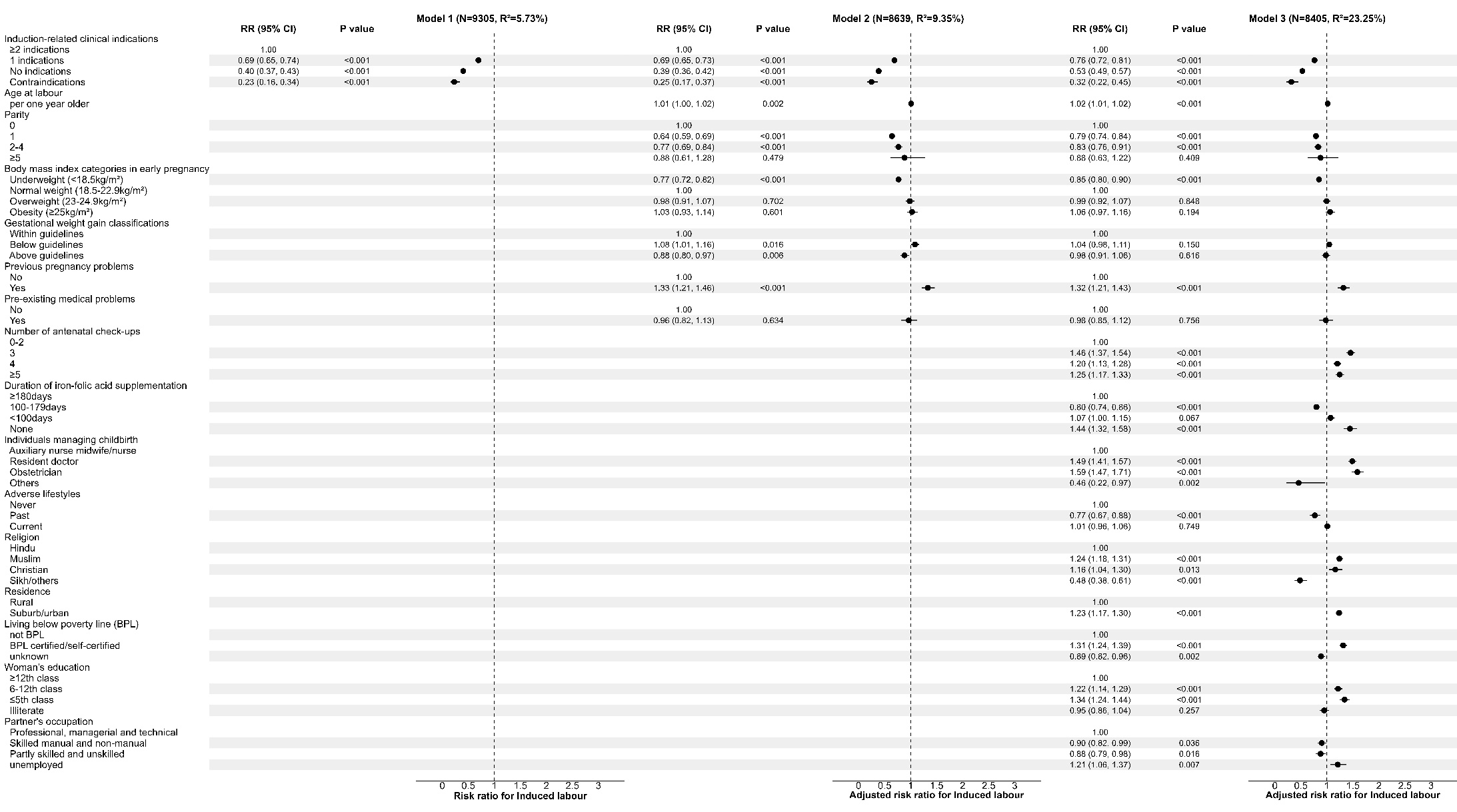


**Supplementary Figure S4: Area under the receiver operating characteristics (AUROC) curve for prediction models for labour induction**


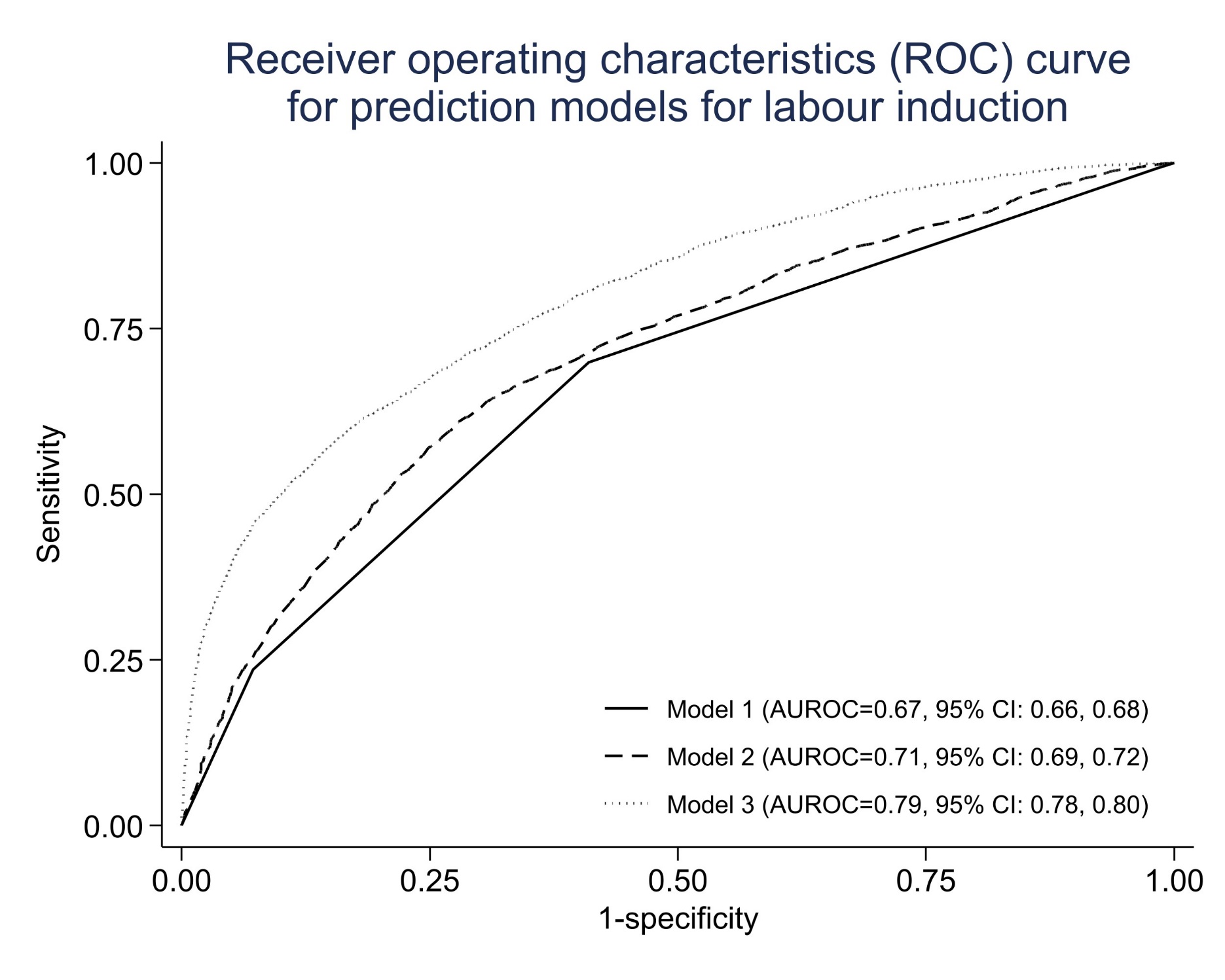


**Supplementary Figure S5: Associations of maternal and household characteristics with labour augmentation in a prospective study in India, expressed as risk ratios**


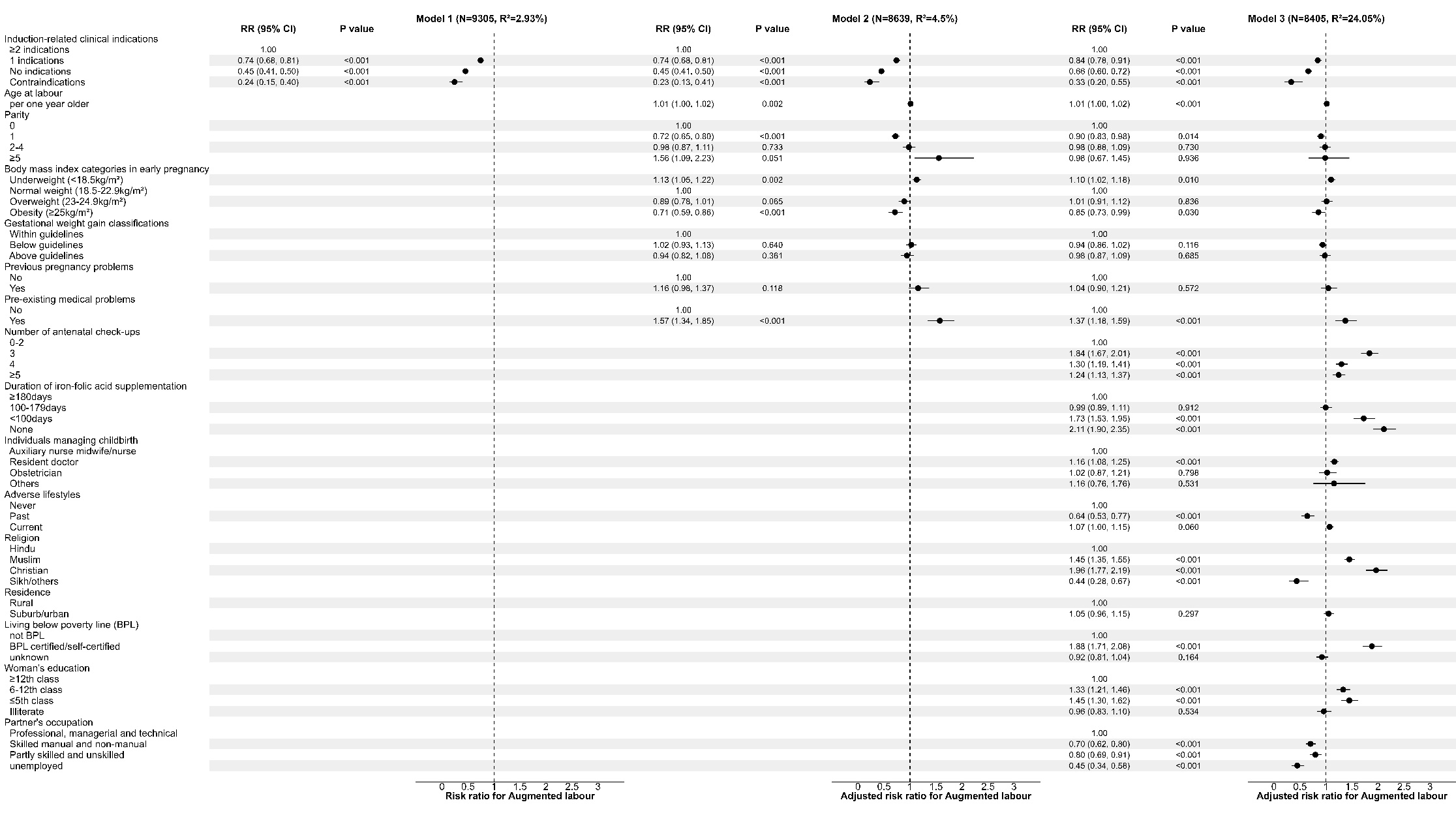


**Supplementary Figure S6: Associations of maternal and household characteristics with labour augmentation, additionally adjusted for labour induction, in a prospective study in India**

**
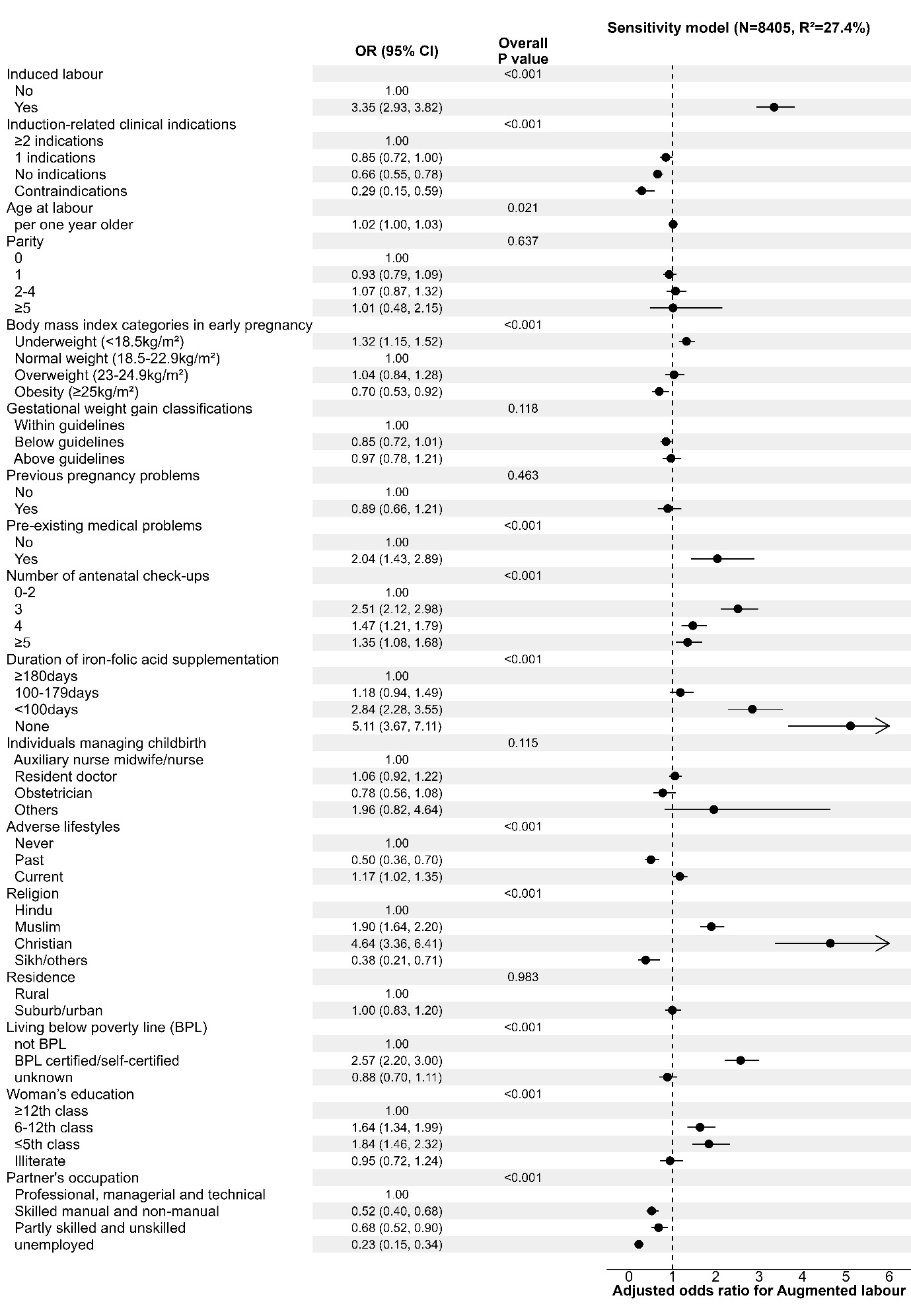
**

**Supplementary Figure S7: Area under the receiver operating characteristics (AUROC) curve for prediction models for labour augmentation**


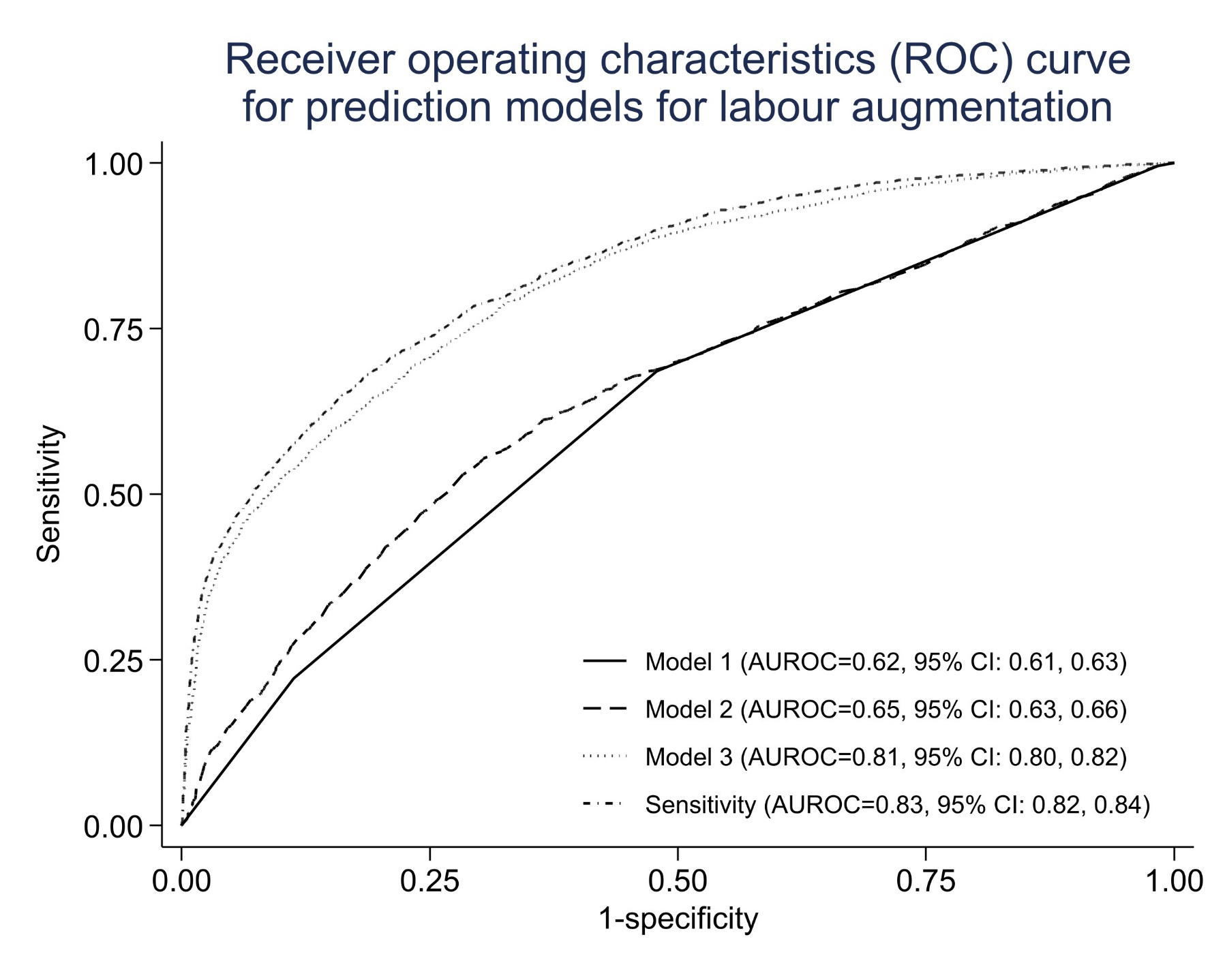


**Supplementary Figure S8: Associations of maternal and household characteristics with labour induction and augmentation, additionally adjusted for COVID-19 period, in a prospective study in India**


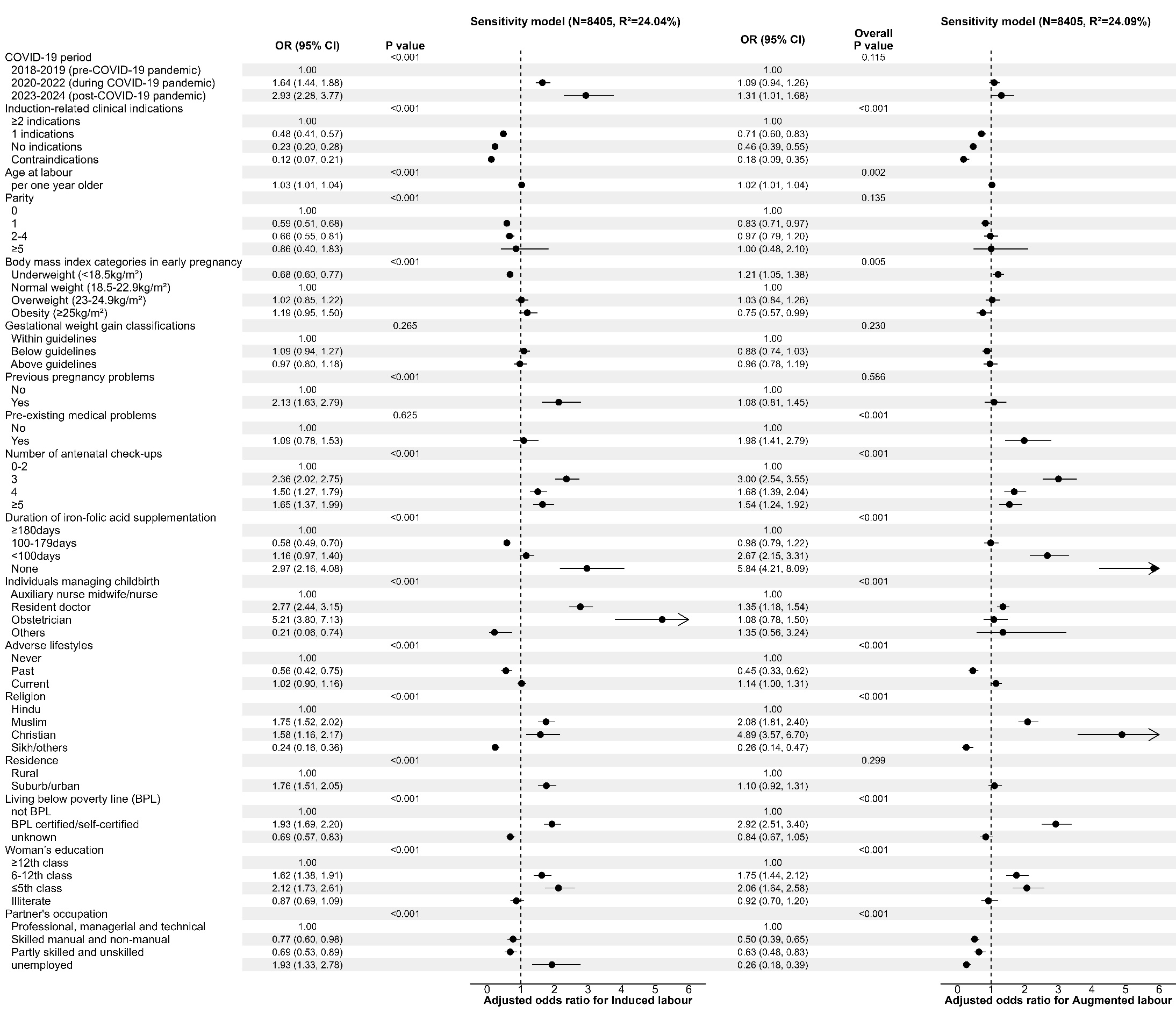


| **Supplementary Table S3: Adjusted associations of socio-economic factors with clinical indications for labour induction and augmentation in a prospective study in India**^‡^ | | | | | | |
| --- | --- | --- | --- | --- | --- | --- |
|  | **Labour induction (n=8405)** | |  | **Labour augmentation (n=8405)** | |  |
|  | **Adjusted OR (95% CI)** | **Overall P value** |  | **Adjusted OR (95% CI)** | **P value** |  |
| No induction/augmentation | No induction (base outcome) | |  | No augmentation (base outcome) | |  |
| Induction/augmentation with ≥1 clinical indications |  |  |  |  |  |  |
| Religion |  | <0.001 |  |  | <0.001 |  |
| Hindu | 1.00 |  |  | 1.00 |  |  |
| Muslim | 2.30 (1.98, 2.67) |  |  | 2.45 (2.09, 2.88) |  |  |
| Christian | 1.66 (1.16, 2.37) |  |  | 4.51 (3.09, 6.57) |  |  |
| Sikh/others | 0.26 (0.16, 0.43) |  |  | 0.16 (0.06, 0.43) |  |  |
| Residence |  | <0.001 |  |  | 0.857 |  |
| Rural | 1.00 |  |  | 1.00 |  |  |
| Suburb/urban | 1.64 (1.38, 1.95) |  |  | 0.98 (0.79, 1.22) |  |  |
| Living below poverty line (BPL) |  | <0.001 |  |  | <0.001 |  |
| not BPL | 1.00 |  |  | 1.00 |  |  |
| BPL certificate/self-certified | 2.20 (1.90, 2.55) |  |  | 3.19 (2.65, 3.85) |  |  |
| unknown | 0.99 (0.80, 1.22) |  |  | 1.14 (0.87, 1.49) |  |  |
| Woman’s education |  | <0.001 |  |  | <0.001 |  |
| ≥12th class | 1.00 |  |  | 1.00 |  |  |
| 6-12th class | 2.18 (1.79, 2.64) |  |  | 2.27 (1.77, 2.92) |  |  |
| ≤5th class | 3.51 (2.78, 4.43) |  |  | 3.27 (2.47, 4.32) |  |  |
| Illiterate | 1.37 (1.05, 1.78) |  |  | 1.48 (1.06, 2.05) |  |  |
| Husband’s occupation |  | <0.001 |  |  | <0.001 |  |
| Professional, managerial and technical | 1.00 |  |  | 1.00 |  |  |
| Skilled manual and non-manual | 0.92 (0.69, 1.24) |  |  | 0.64 (0.46, 0.89) |  |  |
| Partly skilled and unskilled | 0.86 (0.63, 1.17) |  |  | 0.81 (0.57, 1.14) |  |  |
| Unemployed | 2.63 (1.76, 3.92) |  |  | 0.49 (0.31, 0.79) |  |  |
| Induction/augmentation with non-/contra-indication |  |  |  |  |  |  |
| Religion |  | <0.001 |  |  | <0.001 |  |
| Hindu | 1.00 |  |  | 1.00 |  |  |
| Muslim | 1.14 (0.93, 1.40) |  |  | 1.58 (1.28, 1.96) |  |  |
| Christian | 1.35 (0.89, 2.03) |  |  | 4.61 (3.12, 6.79) |  |  |
| Sikh/others | 0.26 (0.15, 0.44) |  |  | 0.45 (0.22, 0.94) |  |  |
| Residence |  | <0.001 |  |  | 0.004 |  |
| Rural | 1.00 |  |  | 1.00 |  |  |
| Suburb/urban | 1.97 (1.63, 2.37) |  |  | 1.43 (1.12, 1.83) |  |  |
| Living below poverty line (BPL) |  | <0.001 |  |  | <0.001 |  |
| not BPL | 1.00 |  |  | 1.00 |  |  |
| BPL certificate/self-certified | 1.71 (1.44, 2.02) |  |  | 2.51 (2.02, 3.13) |  |  |
| unknown | 0.43 (0.32, 0.57) |  |  | 0.47 (0.32, 0.67) |  |  |
| Woman’s education |  | <0.001 |  |  | <0.001 |  |
| ≥12th class | 1.00 |  |  | 1.00 |  |  |
| 6-12th class | 1.21 (0.99, 1.47) |  |  | 1.31 (1.02, 1.68) |  |  |
| ≤5th class | 1.03 (0.79, 1.36) |  |  | 0.95 (0.69, 1.30) |  |  |
| Illiterate | 0.56 (0.40, 0.76) |  |  | 0.52 (0.35, 0.76) |  |  |
| Husband’s occupation |  | 0.038 |  |  | <0.001 |  |
| Professional, managerial and technical | 1.00 |  |  | 1.00 |  |  |
| Skilled manual and non-manual | 0.70 (0.53, 0.94) |  |  | 0.45 (0.33, 0.62) |  |  |
| Partly skilled and unskilled | 0.66 (0.48, 0.91) |  |  | 0.53 (0.38, 0.74) |  |  |
| Unemployed | 0.49 (0.27, 0.89) |  |  | 0.09 (0.04, 0.19) |  |  |
| ^‡^Socio-economic factors mutually adjusted and additionally adjusted for age at labour, parity, body mass index in early pregnancy, gestational weight gain, previous pregnancy problems, pre-existing medical problems, number of antenatal check-ups, duration of iron-folic acid supplementation, healthcare professional managing childbirth and adverse lifestyles. | | | | | | |
